# History of the streptothricin antibiotics and evidence for the neglect of the streptothricin resistome

**DOI:** 10.1038/s44259-023-00020-5

**Published:** 2024-02-07

**Authors:** Ezabelle Franck, Terence S. Crofts

**Affiliations:** Department of Biomedical Sciences, Florida State University, Tallahassee, FL USA

**Keywords:** Antimicrobial resistance, Antibiotics, Antibacterial drug resistance

## Abstract

The streptothricin antibiotics were among the first antibiotics to be discovered from the environment and remain some of the most recovered antimicrobials in natural product screens. Increasing rates of antibiotic resistance and recognition that streptothricin antibiotics may play a role in countering so-called super-bugs has led to the re-evaluation of their clinical potential. Here we will review the current state of knowledge of streptothricins and their resistance in bacteria, with a focus on the potential for new resistance mechanisms and determinants to emerge in the context of potential widespread clinical adoption of this antibiotic class.

## Introduction

Antimicrobial-resistant infections are estimated to cause 700,000 deaths globally each year. By 2050, it is projected that the annual death toll will increase to 10 million and cost the global healthcare system in excess of $100 trillion^1^. Meanwhile, there has been a drought in the development of new antimicrobial therapeutics^2^, especially from major pharmaceutical companies which normally drive antibiotic development^3,4^. One solution to the dual problems of resistance and an anemic antimicrobial pipeline is to re-examine previously discarded antibiotics. Daptomycin, vancomycin, and colistin were previously considered to be too toxic for clinical use in the context of, at the time, sufficient coverage by other antibiotics such as β-lactams^2,5–10^. Increased resistance to first-choice antibiotics led to renewed interest in these more toxic options, and the side effects that kept daptomycin, vancomycin, and colistin off the market were moderated through changes in dosing, increasing purity, and altering pharmacokinetics^9–11^. These formerly discarded antibiotics are now critical medicines in the treatment of multidrug-resistant infections that would otherwise be fatal^12,13^. Similarly, many antimicrobials recently approved by the FDA are derivatives of compounds that were discovered decades ago, such as plazomicin (a derivative of the aminoglycoside antibiotic sisomycin), lefamulin (a semi-synthetic derivative of the antibiotic pleuromutilin), and the tetracyclines eravacycline and omadacyline (third generation tetracycline antibiotics)^14^.

It is in this context that interest in streptothricin antibiotics, one of the first discovered antimicrobial classes but one that has been dogged by toxicity issues, has been renewed. Recent work by Dowgiallo et al. and Morgan et al. has highlighted the clinical potential of streptothricins in combatting multidrug-resistant pathogens by suggesting new routes to diversify streptothricins for increased clinical efficacy and by elucidating their molecular targets^15,16^. Given this reinvigoration of the field, it is worth re-visiting the current state of knowledge of bacterial resistance to streptothricins in the context of their potential therapeutic future. Here, we review important milestones in streptothricin history, its biochemistry and mechanism of action in susceptible bacteria, and the current state of microbial resistance to streptothricins. Our goal is to highlight the apparent disconnect between the abundance of streptothricins in the soil environment and the paucity of mechanisms for their resistance in the soil resistome (the collection of resistance genes and their precursors in an environment), especially compared to other natural product antibiotics^17^. We propose that parallel to investigating the medicinal potential of streptothricins, the streptothricin soil resistome must be studied and quantified so that this knowledge can be incorporated into any next-generation streptothricin analogs that may eventually reach the clinic.

## Streptothricin history and biochemistry

### Discovery of streptothricin

**Fig. 1:**
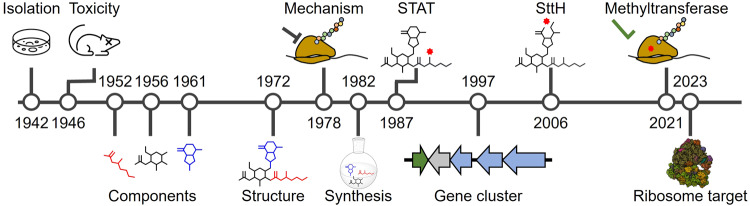
Timeline of streptothricin advancements. 1942: initial discovery, 1946: detailed report of major toxicity during animal testing, 1952–1961: characterization of the main molecular components, 1972: complete structural description, 1978: mechanism of action described, 1982: first total synthesis completed, 1987: first genetic isolation of a streptothricin acetyltransferase (STAT), 1997: resistance-guided discovery of the biosynthetic gene cluster, 2006: first isolation of a streptothricin hydrolase (SttH), 2021: first genetic characterization of a putative streptothricin resistance rRNA methyltransferase, 2023: discovery of the precise molecular targets of streptothricins.

The mass production and clinical deployment of penicillin in the 1940s highlighted the potential for life-saving drugs to come from the soil environment. Selman Waksman and colleagues at Rutgers University sought to make systematic what Alexander Fleming found by serendipity: a procedure for studying the ability of extracts from soil-dwelling bacteria to inhibit pathogenic bacteria, now referred to as the Waksman platform^18,19^. As early as 1940 this approach identified a soil actinomycete that produced a compound capable of killing *E. coli*^19^ and resulted in the purification of another compound, actinomycin, with activity against Gram-positive pathogens^20^. The Waksman platform fully blossomed by 1944 with the discovery of streptomycin, the first anti-tuberculosis antibiotic, and continued to provide additional discoveries^18,21^. In 1942, prior to the more famous streptomycin, Waksman and Woodruff discovered an antibiotic they termed streptothricin, produced by a *Streptomyces lavendulae* isolate (Fig. 1, 1942)^22^. Streptothricin was met with substantial optimism as it appeared to be the first broad-spectrum antibiotic, meaning it was able to kill both Gram-positive and Gram-negative bacteria. This spectrum of bactericidal activity was much wider than that of penicillin, allowing streptothricin to potentially meet a vital healthcare need^22,23^. Initial tests appeared to support this optimism as streptothricin treatment cured *Brucella abortus* model infections of chicken eggs and guinea pigs^24^ and protected mice against a number of Gram-negative pathogens^25^.

### Structure of streptothricins and streptothricin-like compounds

**Fig. 2:**
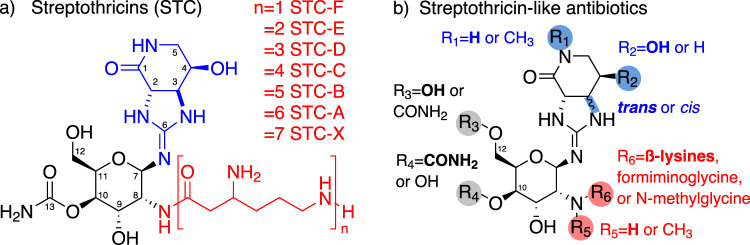
Structures of streptothricin family antibiotics. **a** General structure of the streptothricins (STC) highlighting the streptolidine lactam ring (blue), gulosamine sugar (black), and β-lysine residue(s) (red). Streptothricins are defined by the length of their β-lysine homopolymer chain, from one to seven residues. **b** Structural elements of the “streptothricin-like” antibiotics, highlighting variation in the streptolidine lactam ring (blue: methylation, hydroxylation, stereochemistry), the sugar (black: carbamoyl location), and the amino acid residue (red: N-methylation, presence of β-lysine or a glycine derivative). R-group features in bold signify the default substituent found in canonical streptothricins.

Structurally, the streptothricins are defined by three key molecular features: a streptolidine lactam ring (Fig. 2a, blue component), a gulosamine sugar (Fig. 2a, black component), and a β-lysine homopolymer with lengths of a single β-lysine to seven residues (Fig. 2a, red component)^26–28^. These three core components were identified following chemical degradation of streptothricins^29–32^ (Fig. 1, 1952, 1956, 1961) and confirmed the hypothesis that streptothricin antibiotics (also known then as yazumycins and racemomycins) differ primarily by how many β-lysine residues are attached to the gulosamine sugar^33,34^. The diversity of congeners differing only by β-lysine homopolymer length led to the proliferation of synonyms for streptothricins and streptothricin mixes, including nourseothricin, zhongshengmycin, streptolin, racemomycin, geomycin, pleocidin, yazumycin, phytobacteriomycin, grisein, and polymycin^26^. By convention, individual streptothricins (A–E, X) are identified by the number of β-lysine residues they contain (Fig. 2a). In contrast, the commercially available mixture of streptothricin congeners termed nourseothricin contains streptothricin D (29.6%), E (trace), and F (65.5%)^15,16,35^. In 1972 Khokhlov and Shutova declared that the chemical structure of the streptothricins was fully understood, followed a decade later by confirmation through total synthesis of streptothricin F (Fig. 1, 1972, 1982)^26,27^.

Following the initial description by Waksman and Woodruff^22^, several additional streptothricin-like analogs were identified that strayed from the three central components outlined above. These compounds have historically been termed “streptothricin-like” (Fig. 2b). Streptothricin-like antibiotics with variation in the streptolidine ring include compounds with N-methylation and loss of the C-4 hydroxyl group in albothricin (with compounds A37812 and N-methyl-Streptothricin-D sharing N-methylation without hydroxyl group loss)^36–38^, lactam ring opening in the streptothricin acids^39,40^, or stereocenter inversions in the *cis*-fused streptothricins^41,42^. A number of streptothricin-like compounds also vary at the gulosamine sugar position, most commonly in changes to the location of the carbamoyl moiety from C-10 in the classic streptothricins to C-12 in a variety of analogs^41–43^. Finally, a number of streptothricin-like molecules have been identified that contain non-lysine amino acid residues linked to the gulosamine sugar, including formiminoglycine and N-methylglycine (sarcosine) (*e.g*., compounds LL-AB664, LL-BL136, LL-AC541, sclerothricin, BD-12, BY-81, citromycin, E-749-C, SF-701, A-269A, and A-269A’)^44–52^. It has been estimated that across all these combinations, there are at least 45 streptothricin or streptothricin-like metabolites recorded in the literature^53,54^.

### Streptothricin target and mechanism of action

The aqueous solubility of streptothricins and the presence of an amino sugar in their structure has sometimes led to them being categorized with the aminoglycoside antibiotics. While not accurate, fortuitously the streptothricins do to share a similar mechanism of action as the aminoglycosides. Early studies of ^14^C-leucine and ^32^P-phosphate incorporation in bacterial cells suggested that streptothricins target the bacterial translation apparatus while not affecting DNA synthesis or general cellular integrity^55^. Important details on the molecular mechanism of action of the streptothricins were reported by Haupt et al. beginning in 1978 with the observation that, in addition to generally inhibiting protein synthesis, streptothricin F leads to misreading of the mRNA message, making the streptothricins miscoding antibiotics similar to kanamycin and several other aminoglycosides^56^ (Fig. 1, 1978). A series of in vitro translation assay systems demonstrated that streptothricin F moderately inhibited binding of charged tRNAs to the ribosome but did not inhibit ribosome peptidyltransferase activity. Instead, the greatest effect of streptothricin F on translation appeared to come *via* blocking of the translocation reaction^57^. More recently, Morgan et al. used cryogenic electron microscopy (cryo-EM) to solve the structure of streptothricins F and D bound to their molecular target on the *Acinetobacter baumannii* ribosome (Fig. 1, 2023). They found that the antibiotics principally bind at helix 34 of the 16 S rRNA, specifically interacting with bases A1196, C1054, and U1052 (*E. coli* numbering conventions). The authors hypothesized that these interactions may stabilize noncognate tRNAs in the A-site, leading to streptothricin’s antimicrobial effects^16^. The cryo-EM model also revealed additional binding sites on the ribosome, unique to either streptothricin F or streptothricin D. The authors hypothesized that these could reflect nonspecific binding due to the high concentration of the antibiotics used in the study, but noted that the streptothricins have been proposed to have multiple binding sites on the bacterial ribosome, similar to some aminoglycosides^16,56,58^.

### Streptothricin use in industry, agriculture, and medicine

The broad-spectrum activity and unique molecular mechanism of action suggest streptothricins should be useful tools in the clinical antibiotic armamentarium. However, soon after wider laboratory testing was undertaken it became evident that the streptothricins have appreciable toxicity in mammals. In an early study in rabbits, intravenous, intradermal, oral, and topical applications resulted in organ failure and death (Fig. 1, 1946)^59^. Replicable delayed toxicity of streptothricin and streptothricin-like antibiotics in mammalian test organisms (principally mice) led to delayed toxicity becoming an identifying feature of the whole antimicrobial class^33,34,36,46,49,52,60^. In an effort to characterize this delayed toxicity, Inamori et al. carried out extensive toxicity tests of streptothricins in mice and rats^60–63^. They found that following intravenous administration in mice, streptothricin F distributes largely to the kidneys with very little active antibiotic recoverable from the urine^60^. Histological examination of kidneys from treated mice and rats confirmed streptothricin-induced nephrotoxicity, most notably in the renal cortex, which developed ~48 h after administration^61,62^. Before this delayed toxicity was recognized, the pharmaceutical company Merck attempted a clinical trial of streptothricin in humans which resulted in all four volunteers losing their ability to urinate. The apparent kidney failure was reversible and all four eventually recovered^64^, though this toxicity and the discovery of less toxic broad-spectrum antibiotics resulted in a loss of interest in clinical applications for the streptothricins.

Strategies to mitigate streptothricin toxicity have been partially successful. Treatment of streptothricin D with a streptothricin hydrolase (see section below) was found to result in a 32-fold decrease in activity against *E. coli* but also a larger 128- to 256-fold decrease in toxicity in eukaryotic cells, suggesting a potential medicinal chemistry approach to combating the drug’s side effects^40^. Furthermore, with the discovery that streptothricins vary at the β-lysine position^34^ (Fig. 2a), it was observed that streptothricin toxicity in mice increases with β-lysine chain length. Streptothricin F (one β-lysine residue) was shown to be significantly less toxic than streptothricin D (three β-lysine residues), with reported LD_50_ values of 300 mg/kg vs <10 mg/kg respectively^34^. It has been suggested that the lower cytotoxicity of streptothricin F compared to other streptothricins could reflect decreased cellular internalization by host cells. Takuechi et al. noted that bacterial polycationic isopeptides can directly penetrate mammalian membranes to reach the cytosol. They proposed that the higher toxicity of streptothricins A–E and X compared to streptothricin F may be due to greater host cellular uptake as a result of their longer β-lysine homopolymer tails (Fig. 2a)^65^. A result of this difference in cytotoxicity is that pure streptothricin F, as opposed to a mix of streptothricins, may have a wide enough therapeutic window to justify its use against multidrug-resistant pathogens. Pure streptothricin F has been found to have high activity against pathogens identified by the Center for Disease Control and Prevention to be threats, including vancomycin-resistant *Staphylococcus aureus*, multidrug-resistant *A. baumanni*, and β-lactam resistant Enterobacteriaceae (including the pan drug-resistant *Klebsiella pneumoniae* strain AR-0636), justifying its potential clinical utility^12,15^. This proposition was tested recently by Morgan et al. in a neutropenic mouse model of *A. baumanni* infection where a single dose streptothricin F treatment was associated with no or minimal toxicity and a ~10,000-fold decrease in pathogen titer^16^. This development suggests that streptothricins may follow a similar clinical course to vancomycin where the obstacle of high toxicity was surmounted by increasing antibiotic purity. A recently reported total synthesis of streptothricin F may also lower barriers to the preparation of synthetic streptothricin analogs and allow medicinal chemists to directly tackle toxicity at a molecular level^15^.

Due to their toxicity, streptothricins have not been used in clinical settings, with the possible exception of a 1945 correspondence reporting their use in a balm for treating athlete’s foot^66^. Instead, streptothricins have been predominantly adopted as tools for biotechnology, in particular the congener mix nourseothricin. Because of its broad biological activity and lack of cross-resistance from other selectable markers, nourseothricin and nourseothricin-resistance cassettes are available for a wide spectrum of experimental organisms, both prokaryotic and eukaryotic, including bacteria, fungi, protozoa, plants, animals, and mammalian cell lines^67–72^.

Outside the laboratory, streptothricin use was largely limited until a period between 1981 and 1989 during which nourseothricin was used as an ergotropic compound in pork husbandry in the German Democratic Republic (GDR). Within one year of this practice beginning, it became possible to isolate streptothricin-resistant *E. coli* from pig rectal swabs, agricultural sewage, and from fecal samples from agricultural workers at those farms. These resistant strains were found to contain plasmids encoding multidrug-resistant transposon cassettes with genes for a streptothricin acetyltransferase (see section below) and an aminoglycoside adenyltransferase^73^. Within two years of agricultural nourseothricin use, *E. coli* carrying plasmid-encoded streptothricin resistance could be found in fecal samples from non-farm associated individuals living in the same village as farms employing the antibiotic as a growth-enhancer. Farm workers and individuals from villages where nourseothricin was not used in this way yielded no *E. coli* strains with this resistance phenotype^74^. These represented the first discovered examples of transferable streptothricin resistance genes. The rapid emergence and spread of these resistance determinants as the result of the solely agricultural use of nourseothricin highlights the connection between agricultural antibiotic use and antibiotic resistance in potential human pathogens. Even after discontinuation of this practice, identical streptothricin resistance cassettes appeared to continue to spread into other pathogenic taxa and on other transposons, including ESKAPE pathogens *Enterococcus faecium* and *A. baumannii*, potentially due to selective pressure on the multidrug resistance cassettes from non-streptothricin antibiotic use^3,75,76^. Within the last few decades, a mix of streptothricins termed zhongshengmycin has entered use in Chinese agriculture as a microbial control agent for crops^77,78^. Based on prior experience with nourseothricin in the GDR and, more recently, with colistin^79^, it is likely that extensive agricultural use will result in the appearance of new mobilizable streptothricin resistance determinants.

### Streptothricin biosynthesis and ecology

**Fig. 3:**
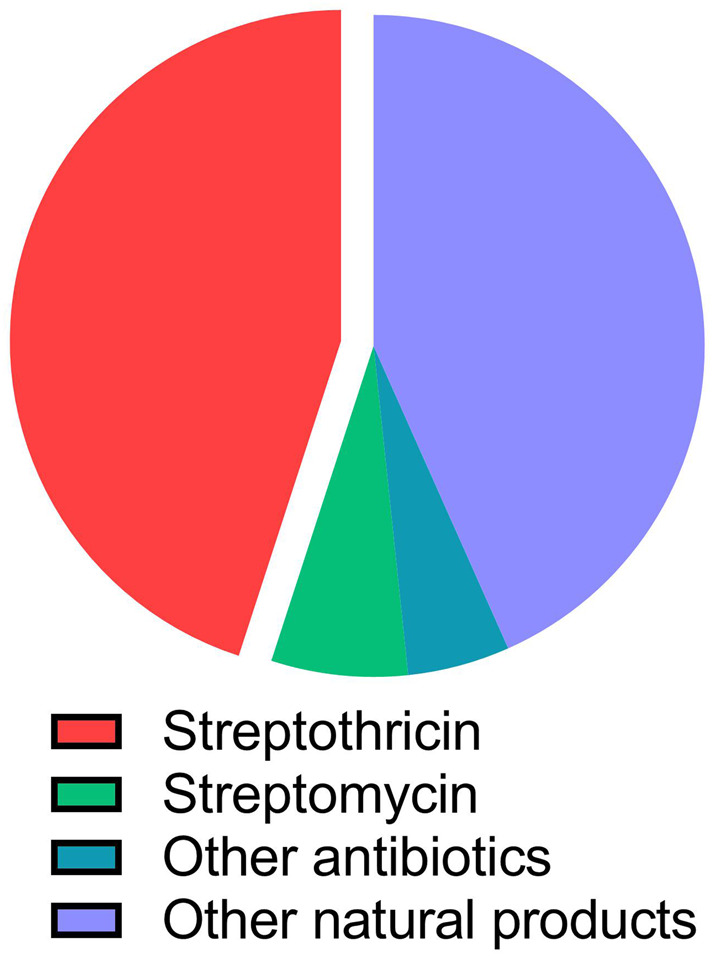
Breakdown of antibiotic production in natural product culture collections. Pie graph charting the prevalence of streptothricin (red), streptomycin (light green), macrolide or tetracycline (dark green), or other natural product (purple) production in an actinomycetes culture collection. Roughly 42% of actinomycetes were found to produce streptothricin. Figure based on data from ref. ^81^.

An interesting aspect of the ecology of streptothricin, and perhaps and explanation for the rapid development of streptothricin resistance in the GDR^73,74^, is the widespread potential for its biosynthesis among soil bacteria. Streptothricin production among actinomycetes bacteria, the taxa most often mined for potential natural product antibiotics^80^, is apparently common. Between 10% and 42% of members of actinomycetes strain libraries have been identified as streptothricin producers across studies (Fig. 3)^18,80–82^. This regularity is supported by the historical record: streptothricin was discovered before any other broad-spectrum antibiotics and was only preceded by the narrow-spectrum compounds penicillin and actinomycin D^22^. The streptothricin biosynthetic gene cluster was discovered in 1997, based on its synteny with a self-protection streptothricin acetyltransferase (Fig. 1, 1997), and this gene cluster has been found across the globe in phylogenetically diverse bacteria^81^. When researchers looked at all publicly available genomes from the *Streptomyces* genus specifically, they found that nearly 4% of genomes encoded a recognizable streptothricin biosynthetic gene cluster^83^. Streptothricins are discovered often enough in natural product screens that methods for streptothricin dereplication have been developed to lower their signal and allow for the discovery of novel compounds^81,84^.

## Bacterial streptothricin resistance mechanisms and determinants

### Overview of the streptothricin resistance landscape

**Fig. 4:**
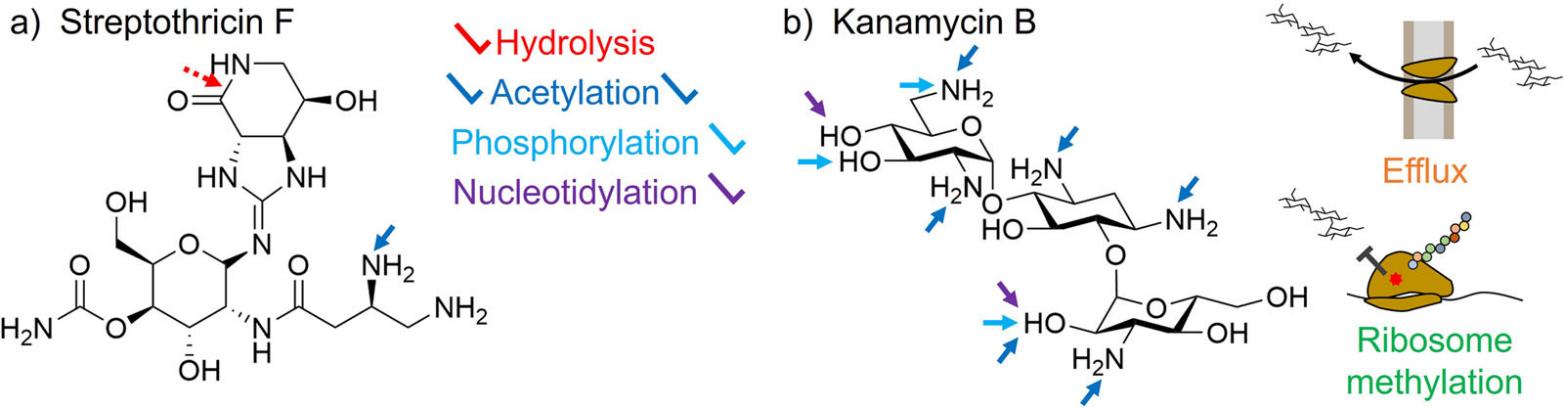
Streptothricin resistance mechanisms compared to kanamycin. **a** Biochemically confirmed streptothricin resistance mechanisms are limited to β-lysine acetylation and (potentially nonspecific) hydrolysis of the streptolidine lactam ring. **b** Kanamycin B resistance mechanisms include acetyl, phosphoryl, and nucleotidyltransferases targeting multiple sites, drug efflux, and 16 S ribosome subunit modification through methylation.

The widespread biosynthesis of streptothricins (Fig. 3) suggests them to be ecologically important compounds in the soil microbiome. One current understanding of antibiotic ecology is that resistance genes are common in the soil microbiome (the resistome) due to the biosynthesis of many natural product antibiotics in that environment^17,79,85^ and that many resistance genes originate from producers of antimicrobials as self-protection genes^85–87^. This understanding of the interactions between antimicrobial production and resistance suggests that a commonly produced compound like streptothricin (Fig. 3) should be associated with widespread resistance in the soil microbiome. However, as described below, streptothricin resistance genes are limited to a few streptothricin acetyltransferases families^88^, two hydrolases for which resistance may be a moonlighting activity^40,89^, and a single putative methyltransferase^90^ (Fig. 4a). This paucity of mechanisms (two to three) and biochemically validated enzymes (single digits across all mechanisms) is in sharp contrast with the resistomes of other antimicrobials. A striking example of this inequality can be found by comparison to the aminoglycoside family of antibiotics. The aminoglycosides were discovered soon after streptothricin and are also characterized by high aqueous solubility, targeting of the 30 S ribosome, and biosynthesis by soil-derived actinomycetes^91^. Taking kanamycin B as a model aminoglycoside, there are at least eight atomic targets for resistance found on the molecule itself, with three different classes of transferases acting at these sites^92,93^. Resistance to kanamycin B and other aminoglycosides is also conferred through active efflux and protection/alteration of the ribosome target^88,94^ (Fig. 4b). While not as extensively modified as aminoglycosides, other ribosome-targeting antibiotics are resisted through significantly more mechanisms as well. For example, chloramphenicol is modified by acetyltransferases (including type A and type B classes encompassing dozens of gene families), hydrolases, oxidases, and nitroreductases and resisted *via* efflux pumps and ribosome methylation^95–100^, tetracyclines are modified by multiple families of tetracycline destructases and are resisted through efflux, ribosome methylation, and ribosome protection^101–103^, and, finally, macrolides are modified both by esterases and phosphotransferases and are subject to efflux, ribosome methylation, and ribosome protection as well^104^.

This inequality in resistance determinants between streptothricins and comparable antibiotics contrasts with what ecological theory would predict given the apparent abundance of streptothricin producers in the soil microbiome. We pose that it is an interesting question whether this imbalance reflects a true paradox or not. One resolution to this paradox is that streptothricins are somehow more immune to bacterial resistance than other antibiotics and are therefore excellent candidates for the clinic. A second resolution is that the lack of known resistance reflects the lack of widespread use (and selection for resistance) and in-depth study, suggesting that if clinical use is pursued it should be coupled with scrupulous monitoring for the transfer of as-yet unknown environmental streptothricin resistance genes into human pathogens. The resolution of this paradox has important implications as streptothricin is re-examined for clinical utility and suggests it would be appropriate to review the current state of knowledge of streptothricin resistance.

### Resistance through drug modification: streptothricin acetyltransferases

**Fig. 5:**
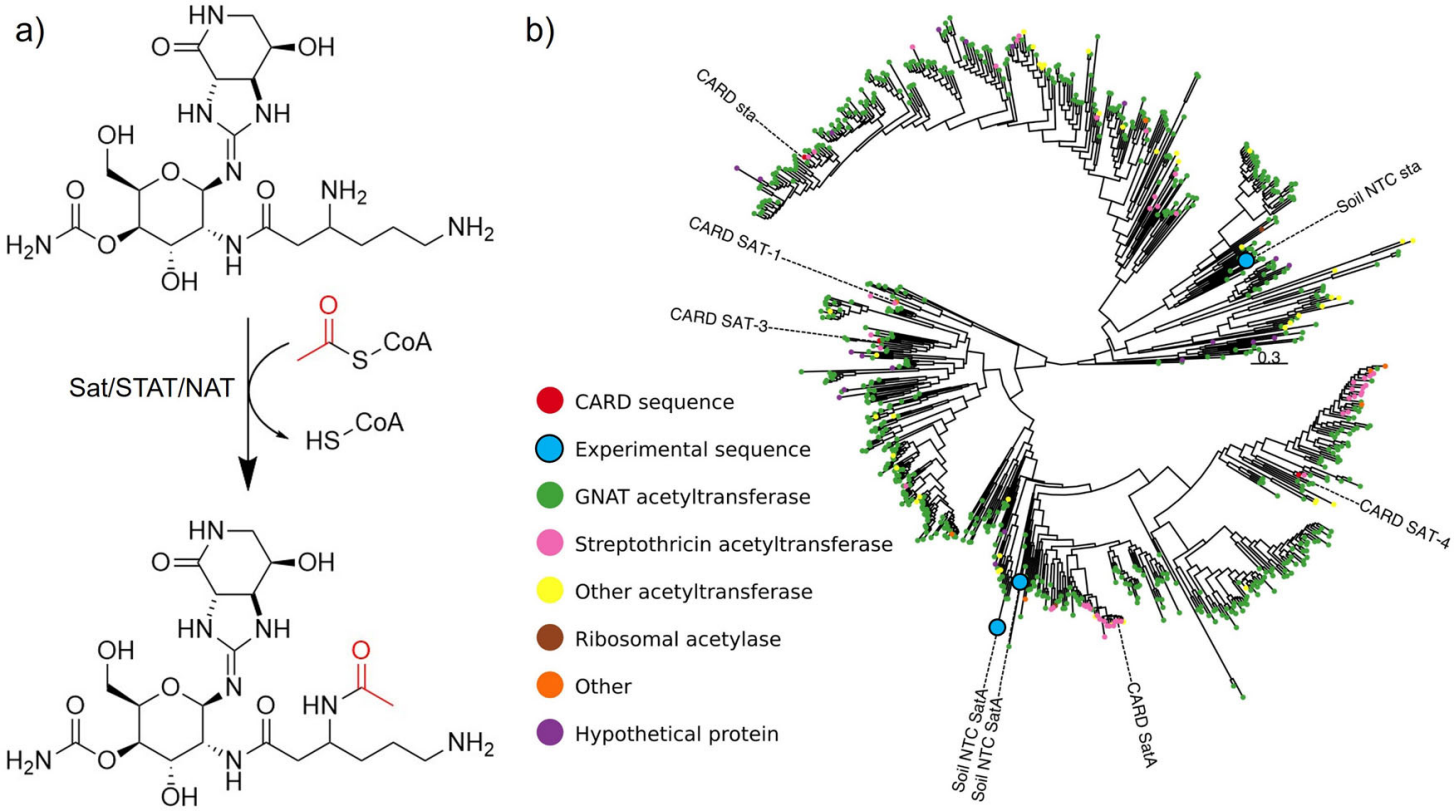
Streptothricin acetyltransferase mechanism and diversity. **a** Streptothricin acetyltransferase (Sat, STAT, or NAT) mechanism of action. Enzymatic transfer of an acetyl group from acetyl-CoA to the streptothricin amino group of β-lysine results in loss of antibiotic activity. **b** Phylogenetic tree of three novel predicted streptothricin acetyltransferases (“Soil NTC SatA/sta”, blue dots) captured by functional metagenomic selection in the context of CARD validated STAT, SatA, Sat-2, Sat-3, and Sat-4 enzymes, predicted streptothricin acetyltransferases, and other related acetyltransferases. **b** is modified from ref. ^90^ (CC BY 4.0).

By far the best-studied streptothricin resistance mechanism is acetylation targeting the β-lysine amino group (Fig. 5a). Acetylation was first identified as a mechanism of resistance in a streptothricin producer in 1983^105^. In the earliest characterization of this mechanism, it was noted that ribosomes from a streptothricin-producing *Streptomyces* strain were susceptible to inhibition by the antibiotic in vitro, suggesting that a self-protection mechanism must exist. In analogy to self-protection transferases in aminoglycoside-producing organisms, the authors screened protein fractions from the producer for streptothricin-inactivation activity. A fraction with this activity was identified that functioned in the presence of acetyl coenzyme A (acetyl-CoA) but not when ATP was substituted, suggesting an acetyltransferase. Incubation of this fraction with acetyl-CoA and kanamycin, neomycin, or chloramphenicol did not result in attenuation of the antibacterial activity of those compounds, demonstrating specificity for streptothricin^105^. Similar experiments on protein fractions from two other producers, *Streptomyces noursei* and *S. lavendulae*, confirmed streptothricin acetyltransferase activity in those organisms as well^106,107^. The gene responsible for this activity, *stat*, was soon identified and sequenced, followed by purification of the active enzyme itself (Fig. 1, 1987)^107,108^ (notably, streptothricin acetyltransferases have been given the following names: STAT, Nat, Sta, and Sat. We propose that Sat become standard moving forward).

Initial in vitro characterization of the STAT enzyme with streptothricin F demonstrated use of acetyl-CoA as a cofactor and the product was confirmed by ^13^C and ^1^H nuclear magnetic resonance (NMR) spectroscopy to be streptothricin F mono-acetylated at the β-amino group of the β-lysine moiety. Reaction kinetics were found to be consistent with Michaelis-Menten models of enzymatic activity and low *K*_M_ values for acetyl-CoA and streptothricin of 69 μM and 2.3 μM, respectively, were physiologically relevant^109^. An additional study found similar results for a Sat-type enzyme from *E. coli*^110^. More recently, detailed in vitro studies performed on the SatA enzyme from *Bacillus* sp. recorded streptothricin acetyltransferase catalytic efficiencies (*k*_*cat*_/*K*_M_) of 5 × 10^6 ^ M^−1^ s^−1^ (*B. subtilis* SatA) and 8.9 × 10^8 ^ M^−1^ s^−1^ (*B. anthracis* SatA), in line with many other *bona fide* antibiotic resistance enzymes^111,112^. Following random and site-directed mutagenesis of the *B. anthracis* SatA, Burckhardt and Escalante-Semerena suggested that a conserved glutamate at position 137 may catalyze the nucleophilic attack on the acetyl-CoA carbonyl and that a cluster of aromatic (Y149, F154, and Y164) and hydrophobic (L136 and A145) residues are important for binding of streptothricin^112^.

The initially discovered streptothricin acetyltransferases from producers of the antibiotic, such as *S. noursei*^113^ and *Streptomyces rochei*^114^, were joined by genes termed “*sat’*” from non-producers such as *Campylobacter coli*^115^, *E. coli*^73,116–118^, and *B. anthracis* and *B. subtilis*^111^. Following the advent of metagenomic sequencing, homologs of these acetyltransferases have been predicted across many bacterial taxa. However, as of July 2023, the UniProt database^119^ contains just three reviewed streptothricin acetyltransferase proteins while the Comprehensive Antibiotic Resistance Database (CARD)^120^ contains just five canonical streptothricin acetyltransferase protein families (STAT, SAT-2, SAT-3, SAT-4, and SatA). In contrast, CARD describes fourteen unique kanamycin AAC(6’) acetyltransferase families alone, suggesting that streptothricin acetyltransferase diversity is under-sampled. Supporting this, in a 1992 survey of streptothricin-resistant bacteria, Smalla et al. found that 77.5% of environmental isolates and 100% of resistant isolates from the soil lacked known acetyltransferase genes as measured by DNA hybridization assays. At most this indicates the presence of other novel streptothricin resistance mechanisms, and at the very least indicates substantial undiscovered acetyltransferase diversity^121^.

**Fig. 6:**
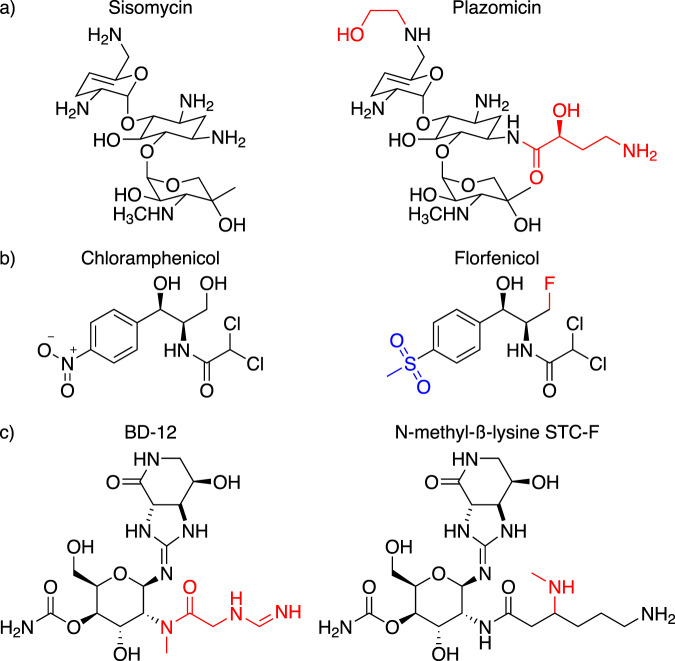
Semi-synthetic antibiotics that resist modification. **a**, **b** are natural product (left) and semi-synthetic (right) antibiotic pairs where modifications (red) have been introduced to combat antibiotic-modifying enzymes. **a** Sisomycin and plazomicin. **b** Chloramphenicol and florfenicol (replacement of the nitro group with a methyl-sulfonyl group, blue, is to combat toxicity, not resistance). **c** BD-12, a formimidoylglycyl-streptothricin and a hypothetical N-methylated streptothricin derivative could potentially avoid streptothricin acetyltransferase inactivation while maintaining activity.

In line with this prediction, we recently proposed a significant increase in Sat enzyme diversity following the use of a functional metagenomic selection for nourseothricin resistance^90^. Briefly, a 162 Gb functional metagenomic library was prepared from soil metagenomic DNA by METa assembly^90^ and housed in *E. coli*. The library was selected by plating cells on agar containing 64 μg/ml nourseothricin and resistance-conferring gene fragments were recovered from colonies arising and were sequenced. Our analyses suggested the screen captured streptothricin acetyltransferases with low to modest amino acid identity to their closest CARD counterparts (26% to STAT, 50% to SatA, and 48% to Sat-4). The phylogenetic distribution relative to canonical streptothricin acetyltransferases from CARD and putative streptothricin acetyltransferase hits strongly suggests that most streptothricin acetyltransferase diversity remains uncharacterized, particularly for the acetyltransferase with 26% identity to STAT (Fig. 5b). If verified by biochemical characterization, this diversity would suggest that streptothricin acetyltransferases are abundant in the soil resistome. The development of next-generation streptothricins for clinical use would benefit from resistance-proofing against these acetyltransferases, much in the same way that the natural products sisomycin and chloramphenicol have been used as templates to prepare semi-synthetic derivatives that are immune to their respective common antibiotic-modifying enzymes. In the case of sisomycin, hydroxyaminobutyryl and hydroxyethyl moieties have been added to primary amines to protect them from modification by a variety of transferases^122^ (Fig. 6a). Chloramphenicol has seen conversion of a hydroxy group into a fluorine to prevent inactivation by acetyltransferases, with the resulting veterinary antibiotic being termed florfenicol^123^ (Fig. 6b). Similarly, modification of the β-lysine target of streptothricin antibiotics could potentially render them immune to modification. The naturally occurring streptothricin analog BD-12 contains a formimidoylglycine group in place of β-lysine(s), notably lacking the β-amino group targeted by streptothricin acetyltransferases^48^. Neither in vitro reactions between BD-12 and a streptothricin acetyltransferase nor the aminoglycoside modifying enzyme AAC(6’)-Ie-APH(6”)-Ia resulted in an acetylated product, suggesting BD-12 may avoid inactivation by acetylating resistance enzymes^124^. Additional streptothricin derivatives with unnatural β-lysine modifications have been prepared through enzymatic modification in vitro, including replacement of β-lysine by 3-aminoproprionyl, 4-aminobutyl, or β-homolysine groups^124,125^. Many of these streptothricin analogs have lower antimicrobial activity compared to the canonical streptothricins, suggesting that incorporating residues more similar to β-lysine, such as N-methylated ones, could potentially retain amine-ribosome electrostatic interactions necessary for high levels of antimicrobial activity (with ribose O2’ groups at positions U1052 and C1054^16^) while avoiding disruptive acetylation by resistance enzymes (Fig. 6c).

### Resistance through drug modification: streptothricin hydrolases

Aside from the streptothricin acetyltransferases, the only other biochemically validated streptothricin resistance enzymes in the literature come from the streptothricin hydrolase family. This enzyme family was first reported in 2006 when Hamano et al. observed that a strain of *Streptomyces albulus* showed greater streptothricin resistance than the streptothricin-producing strain *S. lavendulae* (Fig. 1, 2006). Attempted PCR amplification of *nat* genes from *S. albulus* genomic DNA did not result in any amplicons, suggesting involvement of a novel resistance gene. The authors transformed a streptothricin-sensitive *Streptomyces lividans* strain with a genomic library prepared from *S. albulus* genomic DNA and selected for streptothricin-resistant colonies. Selection of this library resulted in the capture of a 2.9 kb DNA fragment which sequencing revealed to contain three candidate resistance genes. None of the predicted genes showed significant identity to known streptothricin acetyltransferase genes. Genetic dissection of the resistance-conferring fragment revealed a predicted isochorismatase-like hydrolase to be responsible for the observed phenotype^40^. The responsible gene was termed *sttH* and its role in resistance was confirmed by the streptothricin-susceptible phenotype of a *S. albulus sttH* knockout strain and streptothricin-resistant phenotype of *E. coli* expressing *sttH* from a plasmid^40,126^. Using a similar strategy, Maruyama and Hamano captured another resistance-conferring gene from a streptothricin nonproducing *S. noursei* strain. The gene, referred to as *sttH*-sn, came from a homologous region of the *S. noursei* genome and the predicted protein sequence showed 74% identity to *S. albus* SttH^89^.

**Fig. 7:**
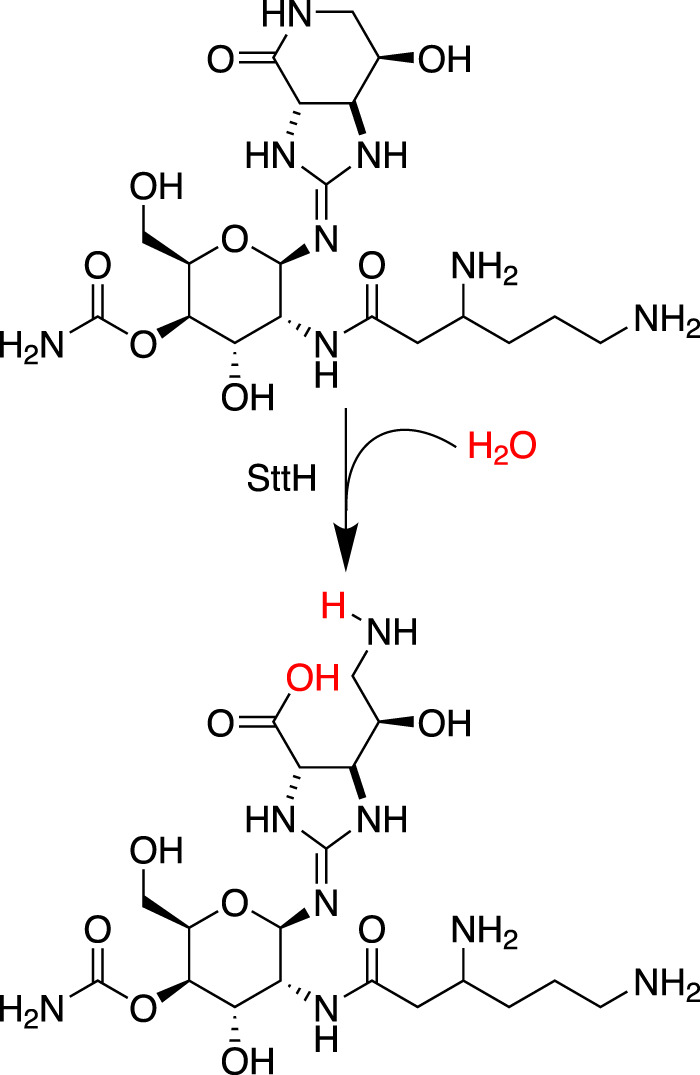
Streptothricin hydrolase mechanism. Streptothricin hydrolase (SttH) catalyzes the opening of the streptolidine lactam ring resulting in an inactive streptothricin acid.

Recombinant SttH and SttH-sn were prepared from *E. coli* and modification of streptothricins F and D was confirmed using liquid chromatography. Mass spectrometry and ^1^H NMR determined that the resulting products were streptothricin acids in which the amide of the streptolidine lactam was hydrolyzed, producing a new primary amine and carboxylic acid (Fig. 7). Streptothricin acids were found by the authors of the study and another group to have significantly lower antimicrobial activity than their corresponding streptothricins, supporting the feasibility of streptolidine hydrolysis as a resistance mechanism^40,43^. Measurement of hydrolysis kinetics demonstrated the two enzymes to be broadly similar in activity, with each showing an apparent slight preference for streptothricin F over D. Interestingly, the *K*_M_ values for streptothricin F and D with both enzymes were measured to be around 1 mM and 3 mM to 6 mM^40,89^, respectively. These *K*_M_ values are orders of magnitude greater than the measured streptothricin F and D minimal inhibitory concentrations of 30 μM and 8 μM for *E. coli*^40^. This *K*_M_, high for an enzymatic reaction where microbial survival is on the line and high compared to SatA (1 μM) and STAT (2.8 μM) enzymes^109,111,112^, suggests that streptothricins are not the native substrates for the SttH enzymes. Supporting this, the authors noted that the genomic context of the two investigated *sttH* genes include open reading frames predicted to function in molybdopterin metabolism, suggesting that the true biological function of the SttH enzymes remains to be determined^89^. The genomic context of *sttH* and high *K*_M_ of the enzyme for its substrate suggest streptothricin hydrolysis is a side activity, making SttH a potential example of a housekeeping enzyme with the ability to evolve into a resistance enzyme^86^.

### Resistance through target modification

**Fig. 8:**
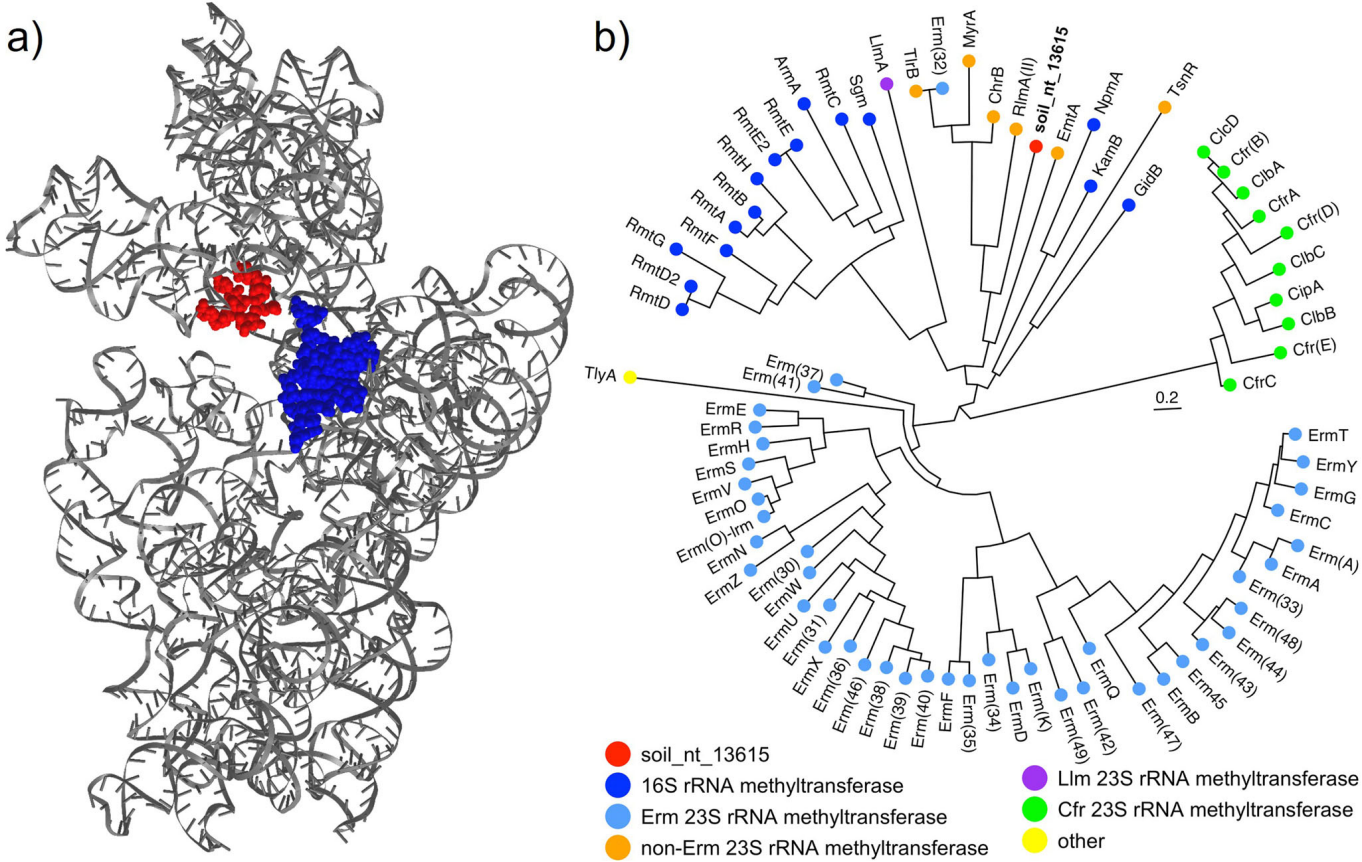
Ribosome modifications conferring streptothricin resistance. **a** *E. coli* 16 S rRNA representation showing approximate regions where mutations confer resistance to kanamycin (space filling, blue) or streptothricin (space filling, red). **b** Phylogenetic tree of ribosome methyltransferases from the comprehensive antibiotic resistance database (CARD) alongside a putative methyltransferase that confers resistance to streptothricin (soil_nt_13615, red). **b** is adapted from ref. ^90^ (CC BY 4.0).

Antibiotics that target the ribosome can also be resisted by protection or modification of the ribosome itself. At its simplest, ribosomal point mutations can decrease antibiotic binding while retaining translational activity. Many aminoglycoside antibiotics can be partially or fully resisted by bacteria with mutations in the helix 44 region of their 16 S rRNA, often in bases 1400–1410 and 1490–1500 (Fig. 8a). In contrast, Morgan et al. found that streptothricin resistance is conferred by mutations (C1054 and A1196) mapping to helix 34 of the 16 S rRNA (Fig. 1, 2023) (Fig. 8a)^16^. Methyltransferase enzymes that methylate specific ribosome bases provide another route to resistance and over a dozen 16 S rRNA methyltransferases are known to confer kanamycin resistance (Fig. 8b, i.e., 16 S rRNA methyltransferases)^88^. In contrast, no biochemically validated methyltransferase enzymes are known to confer streptothricin resistance. Our functional metagenomic selection for nourseothricin resistance, in addition to identifying novel streptothricin acetyltransferases (Fig. 5b), captured a predicted *S*-adenosylmethionine-dependent methyltransferase gene. The predicted novel methyltransferase did not form an outgroup on a phylogenetic tree of rRNA methyltransferase enzymes, but it also did not cluster with the aminoglycoside resistance 16 S rRNA methyltransferases (Fig. 8b). Expression of the methyltransferase-containing DNA fragment in *E. coli* resulted in high-level nourseothricin resistance, shifting the minimal inhibitory concentration for this mix of streptothricins from 4 μg/ml to 1024 μg/ml^90^. Since neither the expression of an Erm 23 S (*ermC*) nor a 16 S (*rmtB*) rRNA methyltransferase conferred streptothricin resistance, it is likely that the novel methyltransferase represents a new class of ribosome methyltransferase. We are currently characterizing the soil_nt_13615 methyltransferase and if biochemical characterization demonstrates its ability to methylate rRNA it would fill one of the missing resistance mechanisms suggested by the comparison of streptothricin to kanamycin (Fig. 4).

### Ecologically predicted resistance mechanisms and future steps

To the best of our knowledge, acetyltransferases targeting the β-amino group of β-lysine (Fig. 5a), streptolidine hydrolases (Fig. 7), ribosome mutation (Fig. 8a), and a putative rRNA methyltransferase (Fig. 8b) are the only documented bacterial resistance mechanisms against streptothricins, despite the apparent abundance of this antibiotic class in the soil microbiome (Fig. 3). To continue the comparison used above, kanamycin resistance mechanisms dwarf that of streptothricin and include acetyltransferases, phosphotransferases, nucleotidyltransferases, and rRNA methyltransferases (each targeting multiple sites), as well as efflux pumps (Fig. 4b). We therefore predict that some, or all, of these mechanisms exist in the soil resistome awaiting discovery. Genome mining for self-protection genes in streptothricin producers, further streptothricin functional metagenomic selections, and comparative genome analyses between resistant and susceptible bacterial taxa offer paths forward to uncovering these predicted mechanisms.

If streptothricins are developed for clinical use in humans, it would behoove medicinal chemists to consider the myriad ways bacteria may develop resistance to the antibiotic and attempt to future-proof second-generation streptothricins against this possibility (Fig. 6c). A thorough cataloging of resistance mechanisms in the soil microbiome that could mobilize into pathogens is the first step of this process, and the divergence between the expected prevalence of streptothricin in the environment and the paucity of resistance mechanisms suggests that significant work remains before this is accomplished.

### Reporting summary

Further information on research design is available in the Nature Research Reporting Summary linked to this article.

## Supplementary information

Reporting Summary
